# Phase I trial of the combination of the pan**-ErbB** inhibitor neratinib and mTOR inhibitor everolimus in advanced cancer patients with ***ErbB* family gene alterations**

**DOI:** 10.1016/j.esmoop.2025.104136

**Published:** 2025-02-04

**Authors:** S.A. Piha-Paul, C. Tseng, H.T. Tran, A. Naing, E.E. Dumbrava, D.D. Karp, J. Rodon, T.A. Yap, K.P. Raghav, S. Damodaran, X. Le, P.T. Soliman, J. Lim, F. Meric-Bernstam

**Affiliations:** 1 Department of Investigational Cancer Therapeutics (A Phase I Clinical Trials Program), University of Texas MD Anderson Cancer Center, Houston, USA; 2 Department of Thoracic, Head and Neck Medical Oncology, University of Texas MD Anderson Cancer Center, Houston, USA; 3 The Sheikh Khalifa Bin Zayed Al Nahyan Institute for Personalized Cancer Therapy, University of Texas MD Anderson Cancer Center, Houston, USA; 4 Therapeutics Discovery Division, University of Texas MD Anderson Cancer Center, Houston, USA; 5 Department of Gastrointestinal Medical Oncology, University of Texas MD Anderson Cancer Center, Houston, USA; 6 Department of Breast Medical Oncology, University of Texas MD Anderson Cancer Center, Houston, USA; 7 Gynecologic Oncology & Reproductive Medicine, University of Texas MD Anderson Cancer Center, Houston, USA; 8 Pharmacy Clinic Programs, Division of Pharmacy, University of Texas MD Anderson Cancer Center, Houston, USA; 9 Department of Breast Surgical Oncology, University of Texas MD Anderson Cancer Center, Houston, USA

**Keywords:** neratinib, everolimus, ErbB-2, ErbB-3, ErbB-4, EGFR

## Abstract

**Background:**

The ErbB family of receptor tyrosine kinases are key targets for antitumor therapy. Although neratinib, a pan-ErbB kinase inhibitor, is approved in ErbB2-positive breast cancer, drug resistance is common. Preclinical data suggest that combining neratinib with the mTOR inhibitor everolimus may overcome such resistance.

**Patients and methods:**

Our trial evaluated this combination’s safety and efficacy in advanced cancers with ErbB alterations. We conducted a phase I dose-escalation trial of neratinib and everolimus. Primary objectives were to assess safety, tolerability, and dose-limiting toxicities (DLTs) and establish the maximum tolerated dose (MTD). Secondary objectives included objective response by RECIST v1.1 and pharmacokinetic analyses.

**Results:**

Twenty-two patients (median age 61, median of four prior therapies) with ErbB alterations (mutations 63.6%, amplification 36.3%, or ErbB2-overexpressed by immunohistochemistry 9.1%) were enrolled. Common tumor types included breast (31.8%), colorectal (18.2%), cervical (9.1%), and endometrial (9.1%) cancers. Frequent grade (G) 3 treatment-related adverse events were diarrhea (18.2%), anemia (9.1%), mucositis (9.1%), and acute kidney injury (9.1%). DLTs included G3 mucositis and diarrhea at dose level (DL) 5, and G3 increased creatinine at DL4. The MTD was DL4: neratinib 240 mg with everolimus 7.5 mg. The objective response rate was 19% with partial response in four patients. Stable disease ≥16 weeks was seen in two patients (9.5%), resulting in a clinical benefit rate of 28.6%.

**Conclusion:**

Pharmacokinetic data indicated reduced neratinib clearance possibly due to CYP3A4 pathway saturation by everolimus. Combination therapy with neratinib and everolimus has a tolerable safety profile and clinical activity in ErbB-altered patients. ErbB family receptors and the PI3K pathway are commonly implicated in oncogenesis. This clinical study of neratinib and everolimus demonstrated favorable clinical activity and tolerability.

## Highlights

• This phase I trial determined the MTD, safety, and efficacy in patients with ErbB alterations.
• ErbB receptors and PI3K pathway drive cancer; neratinib and everolimus showed promising activity and tolerability.
• Combination therapy showed tolerable safety and clinical activity; everolimus may affect neratinib clearance.

## Introduction

The epidermal growth factor receptor family, consisting of four members including epidermal growth factor receptor (EGFR; ErbB1), human epidermal growth factor receptor (HER)-2/ErbB2, HER-3/ErbB3, and HER-4/ErbB4, collaborate to transmit extracellular signals to regulate fundamental cellular processes such as cell growth, metabolism, development, differentiation, and survival.^1^, ^2^, ^3^ Upon activation, the receptors homodimerize or heterodimerize, leading to autophosphorylation within the cytoplasmic domain, and subsequent stimulation of downstream pathways including phosphoinositide 3-kinase/protein kinase B/mammalian target of rapamycin (PI3K/AKT/mTOR).^3^^,^^4^ Overactivation of ErbB signaling can be caused by gene amplification, mutation, or protein overexpression, which lead to pro-oncogenic features such as increased cell survival, resistance to apoptosis, and increased cell invasiveness.^3^ The role of ErbB signaling in oncogenesis is underscored by the prevalence of receptor alterations across various malignancies. For instance, EGFR is overexpressed in over 60% of non-small-cell lung carcinoma (NSCLC)^5^^,^^6^, with mutation rates ranging from 7% to 67% in NSCLC adenocarcinoma patients globally.^7^ Similarly, *ErbB2* mutation exhibits an overall mutation rate of ∼3%, and 2.4% overexpressed by immunohistochemistry (IHC) reported across diverse malignancies.^8^, ^9^, ^10^ *ErbB3* mutations are documented in ∼12% of gastric cancer and 11% of colon cancer, and overexpression of ErbB3 has been reported in multiple tumors.^1^^,^^11^, ^12^, ^13^, ^14^ Despite conflicting evidence of ErbB4 in oncogenesis, *ErbB4* mutations have been reported in melanoma (19%) and overexpressed in colorectal cancer.^14^, ^15^, ^16^, ^17^ The prevalence of these alterations have led to the development ErbB inhibitors as antineoplastic therapies.

Neratinib is a small-molecule pan-ErbB kinase inhibitor that binds irreversibly to the cysteine residues of the tyrosine kinase domain, targeting EGFR/ErbB1, ErbB2, and ErbB4.^18^ *In vitro*, neratinib can effectively decrease cell growth and attenuate autophosphorylation in ErbB2 and EGFR-overexpressing cells.^19^ Additionally, neratinib significantly improved disease-free survival in early-stage ErbB2*-*positive breast cancer, leading to its approval by the US Food and Drug Administration (FDA). In 2017, the FDA approved neratinib for use as extended adjuvant therapy in early-stage ErbB2-positive breast cancer patients who had previously received trastuzumab-based therapy.^20^^,^^21^ By 2020, the FDA extended neratinib’s approval to include the treatment of advanced or metastatic ErbB2-positive breast cancer patients who had received more than two prior anti-ErbB2-based regimens in the metastatic setting, when used in combination with capecitabine.^20^

Despite the promising initial responses seen with neratinib as a monotherapy, its efficacy is often compromised by the activation of alternative signaling pathways, leading to drug resistance. One commonly studied mechanism contributing to this resistance is the activation of the PI3K pathway.^22^^,^^23^ Through a comprehensive genome-wide RNA interference (RNAi) screen, Berns et al. uncovered the pivotal role of *PTEN* in mediating resistance to trastuzumab.^23^ Their findings also revealed that sustained activation of the PI3K pathway, upstream of PTEN, contributes to trastuzumab resistance.^23^ Additionally, investigations by Nagata and colleagues highlighted the significance of the PTEN tumor suppressor in influencing the efficacy of trastuzumab in *ErbB*2-amplified breast cancer.^24^ Retrospective analyses of breast cancer patients receiving combination therapy with trastuzumab and taxane revealed associations between PTEN aberration and diminished responsiveness to trastuzumab, supported by studies in cell cultures and animal models demonstrating the impact of PTEN on reducing the antitumor effects of trastuzumab.^24^

Moreover, Sudhan et al. demonstrated that TORC1, which integrates signaling from PI3K-AKT, is hyperactivated in neratinib-resistant *ErbB2*-mutant cells.^25^ Tumor sequencing data derived from patients revealed an association of aberrant mTOR activation with neratinib resistance.^11^^,^^25^ Sudhan and colleagues further demonstrated that treatment with everolimus, an mTOR inhibitor, was able to overcome resistance by restoring *ErbB2*-mutant tumor cell sensitivity to neratinib. Notably, this restoration of sensitivity was more robust with everolimus than PI3K and MEK inhibitor.^25^ These findings prompted the evaluation of everolimus as a treatment for both *de novo* and acquired resistance to trastuzumab in the phase II studies BOLERO-1 and BOLERO-3.^26^^,^^27^ The results of these studies revealed that patients with ER-negative/ErbB2-positive tumors derived greater benefit from the addition of everolimus.^26^^,^^27^

Our preclinical data further corroborated these findings, indicating that combined therapy of neratinib and everolimus is more effective at suppressing 4E-BP1 and S6 phosphorylation in ErbB2-positive cell lines than neratinib alone.^28^ This combination treatment approach resulted in a notable 100% increase in median event-free survival in 25% of patient-derived xenograft models.^28^

Patients with *ErbB2* alterations often have co-alterations in genes associated with PI3K/AKT/mTOR signaling and multiple trials have explored dual targeting of ErbB2 and the PI3K pathway, including combinations of trastuzumab with buparlisib or copanlisib, yielding preliminary evidence of clinical activity.^29^^,^^30^ We hypothesized that the combination of neratinib and everolimus would improve the efficacy of neratinib in patients with ErbB alterations and that patients with co-alterations would be enriched in enrollment to this study. In this article, we present the safety and efficacy results of a single-center study of neratinib and everolimus in patients with *EGFR* mutations/amplification, *ErbB*2 mutations/amplification, and/or *ErbB*3/4 mutation (NCT03065387).

## Patients and methods

### Study design and dosing

This study was part of an investigator-initiated open-label, single-center, phase I dose-escalation study that utilized a 3 + 3 design for dose escalation^31^ Patients were enrolled into sequential cohorts, and toxicities were evaluated based on the National Cancer Institute Common Terminology Criteria for Adverse Events version 4.0 (CTCAEv4.0). Due to the common occurrence of diarrhea as a side-effect of neratinib, the prophylactic administration of the antidiarrheal medication loperamide was mandatory for all enrolled subjects during the first treatment cycle. The prespecified criteria for dose-limiting toxicity (DLT) included any non-hematologic adverse event of grade (G)3 or 4 that was related to one or more of the study drugs, any G4 hematologic toxicity lasting >1 week; G3 thrombocytopenia with bleeding; neutropenic fever; other G3 non-hematologic toxicity; and any study drug-related severe or life-threatening conditions not defined in the CTCAEv4.0. Certain conditions such as G3 nausea and vomiting that resolved within 72 h with supportive care and optimal antiemetic medical therapy, or G3 diarrhea resolving within 48 h with adequate medical therapy were not considered DLTs. Additionally, alopecia and electrolyte imbalances that resolved with supportive care were not considered to be DLTs. For patients to be considered DLT assessable, they had to have received at least 75% of the study drugs within the first cycle (28 days). Patients who did not complete the first radiographic evaluation would not be assessable for tumor response assessment. The maximum tolerated dose (MTD) was defined as the highest dose at which no more than one of six assessable subjects had a DLT.

The study protocol and amendments were approved by The Institutional Review Board of the University of Texas, MD Anderson Cancer Center. The study was conducted in accordance with the Declaration of Helsinki, Good Clinical Practice, and met all federal, state and local regulatory guidelines. Written informed consent was obtained from all patients before enrollment. The trial registration ID was NCT03065387.

The study was funded by Puma Biotechnology, who also provided neratinib. Everolimus was commercially obtained by patients. Both drugs were administered orally daily on a continuous 28-day cycle, except for the proposed dose level (DL) −1, where everolimus would be taken once every other day. To determine compliance with study, patients were asked to keep pill diaries, and unused drugs were collected at the end of each cycle.

### Eligibility criteria

The study’s key inclusion criteria were advanced solid tumors (not hematologic malignancy) in patients who had failed standard therapy. Patients (irrespective of sex) with pre-identified molecular aberrations predicted to be deleterious, as carried out in the Clinical Laboratory Improvement Amendments (CLIA) environment, were eligible for enrollment, including *EGFR* mutation/amplification, *ErbB2* mutation/amplification, and *ErbB3/4* mutation. Patients with ErbB2-overexpressed by IHC (3+) were eligible if the gene amplification result was unavailable. Other key inclusion criteria were age ≥18 years; tumor lesion size measurable by Response Evaluation Criteria in Solid Tumors (RECIST) v1.1; Eastern Cooperative Oncology Group (ECOG) status ≤1; and sufficient organ functions, including total bilirubin ≤1.5 times of the upper limit of normal (ULN), serum creatinine <1.5 times of ULN, and alanine transaminase (ALT) <2.5 times of ULN (≤5 times of ULN if there were liver metastases). Additionally, absolute neutrophils had to be >1500 cells/ul, platelets ≥100 000/μl, and hemoglobin ≥9 g/dl. All female patients of childbearing potential tested negative for pregnancy.

Key exclusion criteria included patients who were undergoing concurrent chemotherapy or had major surgery within 28 days before everolimus administration, had uncontrolled conditions or illness that could affect their ability to absorb medication or swallow pills (such as gastrointestinal abnormalities), and who required intravenous (i.v) antibiotics for an active infection. Additionally, patients were excluded if they required active treatment with immunosuppressive agents or had significant cardiovascular issues, had active central nervous system metastases or triglyceride level >400 mg/dl, and cholesterol >350 mg/dl.

### Genomic eligibility and analysis

As previously described, patients who had somatic molecular aberrations predicted to be deleterious were identified before study enrollment.^32^ The MD Anderson Precision Oncology Decision Support Services (PODSS) assessed the genomic alterations of potential patients that were relevant to the study. The alterations were compared to published literature, and then categorized based on their variant-level actionability and functional significance, to identify gnomically matched therapies.^32^

The OncoPrint diagram was created using the MCPlotter visualization application, which employs the D-3 JavaScript package and Microsoft’s ASP.NET framework. Mutation data were integrated into the MOCLIA database from pre-existing pathology reports carried out at the MD Anderson Cancer Center or other CLIA-certified facilities. Biomarker data that did not meet the criteria for CLIA mutations or CLIA copy number for external molecular testing reports were manually annotated by the PODSS team. The OncoPrint diagram displays genetic alterations, both pathogenic and variants of unknown significance, observed in patients at the time of study enrollment. Rows represent genes, while columns represent individual patients. Colored cells indicate disease types, cohorts, clinical benefits, and alteration types (e.g. amplification, deletion, duplication, frameshift, missense substitution, overexpression, and truncation).

### Assessment of tumor response

Baseline radiographic imaging was carried out using computed tomography scan or magnetic resonance imaging within 4 weeks of starting treatment. The same imaging techniques were used for subsequent tumor response assessment. Tumor measurements were taken at baseline before treatment and at the end of every two cycles (every three cycles after 24 weeks) until confirmed disease progression or patient removal from study. Additional radiographic imaging was obtained if clinically indicated. All 22 patients who participated in the trial had measurable disease at baseline, assessed according to RECISTv1.1. However, one hemangiopericytoma patient could not complete tumor re-assessment as the patient had discontinued from the trial early due to discovery of a new brain lesion early in cycle 1. As a result, 21 patients were assessable for response. For the purposes of this paper, prolonged stable disease (SD) was defined as lasting ≥16 weeks.

### Statistical considerations

This trial was a non-randomized, phase I dose-escalation study that utilized a 3 + 3 design.^31^ Statistical power calculations were not formally conducted to determine the sample sizes for this study. Instead, it was projected that at least three patients would be included per DL during the accelerated titration phase, and three to six patients per DL in the standard dose-escalation phase, with a minimum of six subjects anticipated to be treated at the MTD level.

### Pharmacokinetics

Pharmacokinetic (PK) analysis was conducted on cycle 1, day 15 for both everolimus and neratinib with blood samples collected at pre-dose, 1, 3, 4, 6, 8, and 24 hours post-dose. Following good clinical and laboratory practices, blood was collected into K2-EDTA vacutainers, gently inverted and mixed, then centrifuged at 3000 rpm (1500 *g*) for 10 min at 4°C. The resultant plasma was placed into cryovials and frozen at −70°C until analysis. Neratinib and everolimus plasma concentrations were quantified using a validated tandem liquid chromatography/mass spectrometry (LC/MS) and PK parameters were determined using noncompartmental analysis methods with the software, Phoenix WinNonlin 8.4 (Certara USA Inc., Princeton, NJ).^33^^,^^34^

## Results

### Demographic and clinical characteristics

Table 1 presents an overview of the baseline demographic and clinical characteristics of the patients who participated in the study on combination therapy with neratinib and everolimus. The study enrolled a total of 22 patients from March 2018 until the data cut-off of 31 July 2023 as shown in the consort flow diagram in Supplementary Figure S1, available at https://doi.org/10.1016/j.esmoop.2025.104136. Patients were enrolled on to five DLs based on a standard 3 + 3 design.^31^ The median age of patients was 61 years, ranging from 41 to 83 years. Seventeen (77.3%) of the enrolled patients were female and 86.4% had an ECOG performance status of 1. Among the different cancer types represented in the study, breast cancer (31.8%, *n* = 7) was the most common, followed by colorectal cancer (18.2%, *n* = 4), cervical cancer (9.1%, *n* = 2), and endometrial cancer (9.1%, *n* = 2).

**Table 1:** Baseline patient demographics and clinical characteristics

Characteristic	NER 160 mg Eve 5 mg	NER 200 mg Eve 5 mg	NER 200 mg Eve 7.5 mg	NER 240 mg Eve 7.5 mg	NER 240 mg Eve 10 mg	All
	*n* = 5	*n* = 4	*n* = 3	*n* = 8	*n* = 2	*n* = 22
Gender, *n* (%)						
Male	2 (40)	1 (25)	0	1 (12.5)	1 (50)	5 (22.7)
Female	3 (60)	3 (75)	3 (100)	7 (87.5)	1 (50)	17 (77.3)
Median age	55.0 (50-69)	50.5 (41-64)	60.0 (43-60)	66.5 (58-75)	65.0 (65-83)	61 (41-83)
ECOG						
0	0	1 (25)	1 (33.3)	1 (12.5)	0	3 (13.6)
1	5 (100)	3 (75)	2 (66.7)	7 (87.5)	2 (100)	19 (86.4)
Tumor type						
Breast	2 (40)	2 (50)	0	2 (25.0)	1 (50)	7 (31.8)
Colorectal	1 (20)	1 (25)	1 (33.3)	1 (12.5)	0	4 (18.2)
Cervical	0	0	1 (33.3)	1 (12.5)	0	2 (9.1)
Endometrium	0	0	0	2 (25.0)	0	2 (9.1)
Esophageal	1 (20)	0	0	0	0	1 (4.5)
Hemangiopericytoma	1 (20)	0	0	0	0	1 (4.5)
Bladder	0	1 (25)	0	0	0	1 (4.5)
Ovarian	0	0	1 (33.3)	0	0	1 (4.5)
Lung	0	0	0	1 (12.5)	0	1 (4.5)
Parotid gland	0	0	0	1 (12.5)	0	1 (4.5)
Unknown primary	0	0	0	0	1 (50)	1 (4.5)
Pan-ErbB alteration (%)						
EGFR amplification	0	0	0	0	0	0
EGFR mutation	1 (20)^a^	1 (25)	0	0	0	2 (9.1)
ErbB-2 amplification						
(FISH)	1 (20)	1^b^ (25)	1 (33.3)^a^	1^b^ (12.5)	0	4 (18.2)
(NGS)	0	1^b^ (25)	2 (66.7)^a^	3^b^ (37.5)^a^	1 (50)	7 (31.8)
ErbB-2 mutation	3 (60)	1 (25)	2 (66.7)^a^	4 (50.0)^a^	1 (50)	11 (50)
ErbB-3 mutation	0	0	0	1 (12.5)	0	1 (4.5)
ErbB-2 (IHC 3+)	1 (20)	2^b^ (50)	0	3^b^ (37.5)	0	6 (27.3)
Number of prior systemic therapies						
Median	3 (2-7)	7 (4-11)	4 (3-6)	5 (2-11)	5 (2-8)	4 (2-11)
Patients with prior ErbB receptors targeted therapy^c^	3 (60)	2 (50)	2 (66.6)	5 (66.6)	1 (50)	13 (59.1)
Patients with prior radiation	2 (40)	2 (50)	1 (33.3)	7 (87.5)	1 (50)	13 (59.1)
Patients with prior PI3K/AKT/mTOR therapy^d^	0	1 (25)	0	1 (12.5)	0	2 (9.1)

D, day; ECOG, Eastern Cooperative Oncology Group; EGFR, epidermal growth factor receptor; ErbB, erythroblastic oncogene B; Eve, everolimus; FISH, fluorescence *in situ* hybridization; IHC, immunohistochemistry; mAb, monoclonal antibody; Ner, neratinib; NGS, next-generation sequencing.

a Co-occurring alterations include the following: EGFR T725M mutation and ErbB2-overexpressed by IHC (*n* = 1, dose level 1); *ErbB2* amplification by NGS and/or FISH and ErbB2 I767M (*n* = 1, dose level 3) and *ErbB2* amplification by NGS and ErbB2 V777L (*n* = 1, dose level 4).

b Patient with *ErbB2* amplification by FISH and/or NGS and ErbB2-overexpressed by IHC (*n* = 1, dose level 2; *n* = 2, dose level 4).

c EGFR-targeted therapy includes investigational agent: KBP-5209, cetuximab, afatinib, and poziotinib; ErbB2-targeted therapies includes trastuzumab, trastuzumab emtansine, pertuzumab, and three investigational agents [an ErbB-2/4-1BB bispecific, ErbB2-targeted bispecific mAb, and an autologous T-cell-expressing T-antigen coupler (TAC) that targets ErbB2].

d PI3K/AKT/mTOR targeted therapy includes: gedatolisib (*n* = 1, dose level 2); everolimus (*n* = 1, dose level 4).

The study included patients with pre-identified pan-ErbB alterations predicted to be activating (Table 1). The most prevalent *ErbB2* alterations were mutation (*n* = 11, 50%) and amplification (*n* = 8, 36.3%). Two patients showed co-occurring alterations, including a colorectal patient with somatic ErbB2 I767M mutation and *ErbB2* amplification confirmed by next-generation sequencing (NGS) and FISH, and a parotid gland patient with *ErbB2* amplification (NGS) and ErbB2 V777L mutation. Among the eight patients with *ErbB2* amplification, one was identified through FISH, five patients by NGS, and two by both by FISH and NGS. Six patients were determined to be ErbB2-overexpressed by IHC. The timeline for identifying ErbB alterations is summarized in Supplementary Table S1, available at https://doi.org/10.1016/j.esmoop.2025.104136.

Throughout the study, the median number of treatment cycles (a cycle lasting 28 days) completed by all patients was 3.5 (range 1-36 cycles). Half of the patients (50%, *n* = 11) received more than two cycles. For patients showing SD or better, the median number of completed cycles was four (range 2-36 cycles). Patients were heavily pre-treated with a median of four prior systemic therapies (range 1-11). Thirteen (59.1%) patients had prior EGFR/ErbB-2 targeted therapy including cetuximab (*n* = 2), lapatinib (*n* = 2), afatinib (*n* = 1), poziotinib (*n* = 1), trastuzumab (*n* = 7), trastuzumab emtansine (*n* = 3), pertuzumab (*n* = 5), trastuzumab deruxtecan (*n* = 3), and four investigational agents: a pan-ErbB agent (*n* = 1), an ErbB-2/4-1BB bispecific (*n* = 3), an ErbB2-targeted bispecific mAb (*n* = 2), and an autologous T-cell-expressing T-antigen coupler (TAC) that targets ErbB2 (*n* = 1). Two patients (9.1%) received prior PI3K and/or mTOR inhibitor including gedatolisib (*n* = 1) and everolimus (*n* = 1).

### Adverse events

Twenty-two patients were administered a combination of neratinib and everolimus in this study, and their safety profile was evaluated. Treatment-related adverse events (TRAEs) are summarized in Supplementary Tables S2 and S3, available at https://doi.org/10.1016/j.esmoop.2025.104136. Among the patients treated on study, 21 patients (95.4%) experienced at least one TRAE. The most common TRAEs (occurring in ≥20%) were: diarrhea (*n* = 14, 63.6%), hypertriglyceridemia (*n* = 11, 50%), mucositis (*n* = 9, 41%), and nausea (*n* = 7, 31.8%). Notably, diarrhea also accounted for the most frequent G3 TRAE and was observed in four patients (18.2%). Additionally, other common G3 TRAEs included mucositis (*n* = 2, 9.1%), anemia (*n* = 2, 9.1%), and acute kidney injury (*n* = 2, 9.1%). Fortunately, no patients experienced G4 or higher TRAEs and no treatment-related deaths were reported on trial.

A total of three DLT events were reported during the study, with two of these events occurring in patients treated at DL 5. These DLTs included G3 diarrhea and G3 mucositis, respectively. As a result of these findings, the previous lower DL (DL 4) was then expanded to include three additional patients, and among them, one patient experienced a DLT of G3 increased creatinine. Therefore, DL 4, which involved continuous daily oral administration of neratinib 240 mg and everolimus 7.5 mg, was designated as the MTD.

In this study, severe adverse events were reported for nine patients. Among these events, only five were attributed to study drug including creatinine increased (*n* = 2), diarrhea (*n* = 2), and overdose on everolimus on cycle 1, day 1 (*n* = 1), as indicated in Supplementary Table S4, available at https://doi.org/10.1016/j.esmoop.2025.104136.

### Efficacy

**Figure 1:**
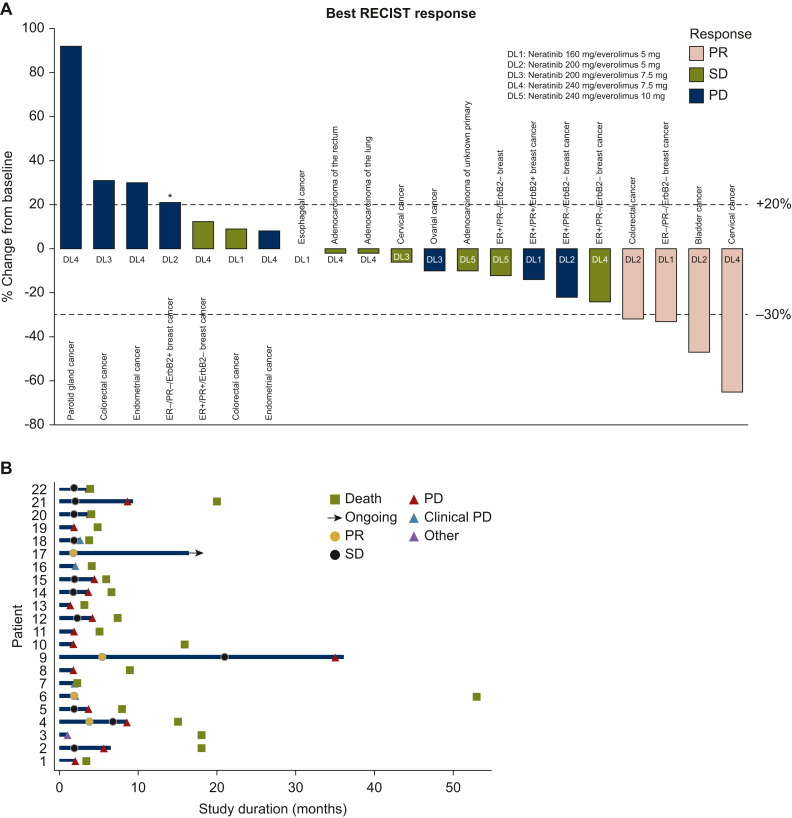
**Antitumor activity and therapy response duration among treated patients.** Waterfall plot (A) and swimmer plot (B) depicting RECISTv1.1 response by patient. (A) Individual patients/cohorts are represented by vertical bars on the *x*-axis. The best RECISTv1.1 response (%) is depicted on the *y*-axis. Twenty-one of 22 patients were assessed for response by RECISTv1.1. One patient was removed from study on cycle 1, day 8 due to discovery of brain metastases on imaging. This patient is not included in the response analysis. One patient was assigned a value of +21% for clinical progression (∗). The upper dotted line indicates progressive disease by RECISTv1.1 (+20% from baseline). The lower dotted line indicates partial response RECISTv1.1 (−30% from baseline). (B) Swimmer plot depicting 22 study participants. Each horizontal line represents a patient. The *x*-axis depicts the duration since treatment commencement, while the *y*-axis represents the patients. DL, dose level; ER, estrogen receptor; ErbB2, avian erythroblastic leukemia viral oncogene homolog 2; PD, progressive disease; PR+/−, progesterone receptor; PR, partial response; SD, stable disease. Note: For breast cancer, ErbB2+ indicates *ErbB2* amplified by FISH/NGS or overexpressed by immunohistochemistry (IHC 3+). ErbB2– indicates ErbB2 negative.

All 22 patients who participated in the trial had measurable disease at baseline, assessed according to RECISTv1.1. However, one hemangiopericytoma patient could not complete tumor re-assessment as the patient had discontinued from trial early due to discovery of a new brain lesion early in cycle 1. As a result, 21 patients were assessable for response and included in the waterfall plot depicted in Figure 1A, which illustrates the best RECISTv1.1 response of patients. Among the 21 patients, there were 4 (19%) partial responses (PRs) and 2 (9.5%) SD ≥16 weeks. A total of 6/21 (28.6%) patients derived clinical benefit [complete response (CR)/PR/SD ≥16 weeks]. As of 31 July 2023, a 63-year-old female patient with adenocarcinoma of the cervix remained active in the study. From the first tumor re-assessment (at 8 weeks), she demonstrated a PR (−37%), achieving a best RECIST response of PR (−65%) by the end of cycle 6. The patient continues therapy and remains in PR, with a duration of response (DOR) of >14 months. The swimmer plot in Figure 1B illustrates the duration of study participation for each patient, with a median duration of 3.2 months (95% confidence interval 2.3-8.7 months). Of the 22 patients enrolled, 21 have discontinued the study. Among these, 72.7% (*n* = 16) were taken off study due to disease progression on radiographic imaging (per RECISTv1.1), including two patients with new brain metastases. Additionally, 13.6% (*n* = 3) of patients experienced clinical progression. One patient discontinued study treatment secondary to treatment-related toxicity of G3 diarrhea, and another patient was removed from study for not meeting protocol criteria, as a new brain lesion was detected on cycle 1, day 8.

### Molecular alterations

**Figure 2:**
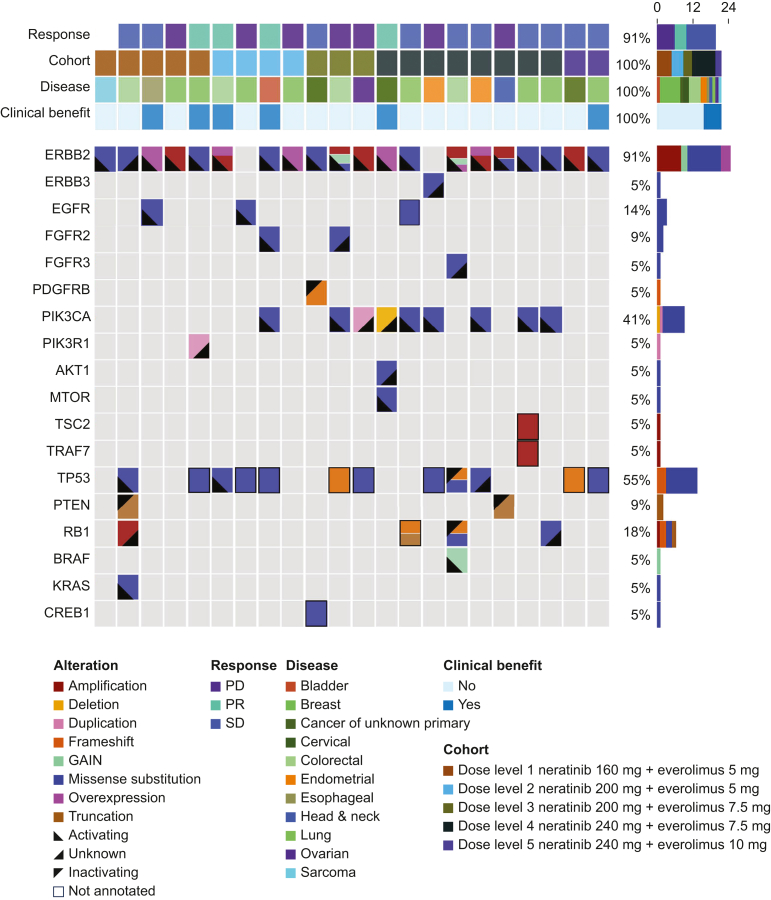
**OncoPrint of genetic alterations in patients.** The Oncoprint diagram illustrates genetic alterations in patients at study enrollment. The row represents genes, and columns represent individual patients. Color cell is used to depict disease types, cohort, clinical benefit, and alteration type (e.g. amplification, deletion, duplication, frameshift, missense substitution, overexpression, and truncation). PD, progressive disease; PR, partial response; SD, stable disease. Note: One patient does not have response data as this patient was taken off study early due to discovery of a brain metastases on imaging carried out on cycle 1, day 8 of therapy. The patient was removed from study as the patient was determined ineligible.

The OncoPrint diagram illustrates the pathogenic ErbB alterations harbored by patients that qualified them for study enrollment. Additionally, it includes co-existing alterations (irrespective of pathogenicity) that are involved in the ErbB downstream pathways, including genes in the MAP kinase pathway (Figure 2). Table 2 shows details on molecular alterations, cohort assignment, duration of treatment, DOR, and best response by RECIST v1.1 in patients that demonstrated clinical benefit. Among the treatment responders listed in Table 2, three patients had *ErbB2* mutations (two PR and one SD), two had ErbB2 amplification or overexpression identified by IHC (two PR and one SD), and one patient had a concurrent of EGFR T725M mutation with ErbB2 overexpression by IHC.

**Table 2:** Stable disease (SD) ≥16 weeks or partial response (PR) by RECISTv.1.1 and characterization by patient

Cancer type	Dose level	Best response RECISTv1.1	No. of prior systemic regimens	No. of prior EGFR/ErbB2-targeted therapy	Response duration (weeks)	Duration of treatment (weeks)	ErbB2 alteration	EGFR mut	RAS mut	Other alterations
TNBC	1	PR (−33%)	2	0	12.7	36.8	ErbB2_S310Y	N	N	PIK3R1_K459_D464dupKSREYD; PMS2_C297Y TP53_T125P PDL1_Postitive
Esophageal	1	SD (0%)	1	1^a^	16	27.9	EGFR T725M ErbB2 IHC 3+	Y	N	*—*
Colorectal	2	PR (−32%)	5	2^b^	66.3	154	*ErbB2* Amplification (FISH/NGS)/IHC 3+	N	N	TP53_G245D
Bladder	2^c^	PR (−47%)	4	0	Unknown^d^	8.7	ErbB2_S310F	N	N	FGFR2_N549K PIK3CA_E545D ATM_R1466^c^ ATM Q264H CDKN2A_N42K TP53_K139N BRCA1_V1234f∗8 BRCA2_E2599K *BRCA2_c.517-1G>C* ERBB2 F864I^e^
Cervical	4	PR (−65%)	2	0	63+	70+	ErbB2 IHC 3+	N	N	mTOR_TI977K AKT1_D323N PIK3CA_L113del
ER+ ErbB2− (IHC 0) breast	5	SD (−12%)	8	0	28.4	40	ErbB2_S310F*;* ErbB2_V777L	N	N	APC G1836fs∗5 TP53 G245S ErbB2_R678Q^e^

Abbreviations: AKT, protein kinase B; APC, adenomatous polyposis coli; ATM, ataxia-telangiectasia mutated; BRCA, breast cancer gene; CDKN2A, cyclin-dependent kinase inhibitor 2A; EGFR, epidermal growth factor receptor; ErbB2, avian erythroblastic leukemia viral oncogene homolog 2; FGFR, fibroblast growth factor receptor; FISH, fluorescence *in situ* hybridization; IHC, immunohistochemistry; mTOR, mammalian target of rapamycin; mut, mutation; N, no; NGS, next-generation sequencing; PIK3CA, phosphatidylinositol-4,5-bisphosphate 3-kinase catalytic subunit alpha; PIK3R1, phosphatidylinositol 3-kinase regulatory subunit 1; PMS2, postmeiotic segregation increased 2; PR, partial response; RAS, rat sarcoma; RECIST, Response Evaluation Criteria in Solid Tumors; SD, stable disease; TNBC, triple-negative breast cancer; TP53, tumor protein 53; Y, yes.

a Trastuzumab.

b Trastuzumab + pertuzumab and ErbB-2 X 4-1BB bispecific.

c This patient was a dosing error as he took everolimus 10 mg p.o. daily instead of 5 mg p.o. daily during cycle 1 of therapy.

d Patient discontinued due to toxicity.

e This mutation is a variant of unknown significance.

### Pharmacokinetics

**Figure 3:**
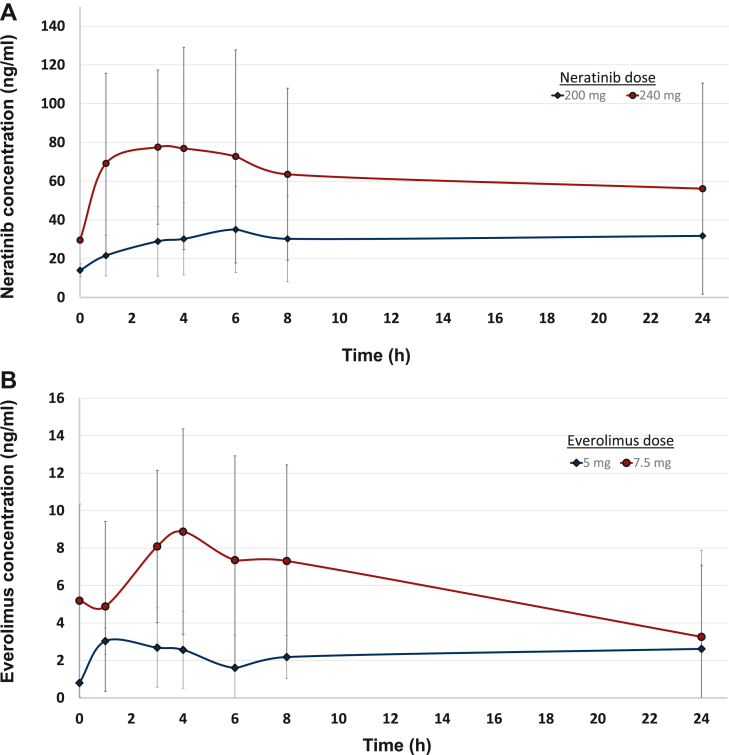
**Plasma concentration profiles of (A) neratinib and (B) everolimus on cycle 1, day 15 of therapy.** Mean plasma concentrations over time on cycle 1, day 15 after co-administration of (A) neratinib at 200 mg (*n* = 3) or 240 mg (*n* = 5) daily and (B) everolimus at 5 mg (*n* = 3) or 7.5 mg (*n* = 5). Standard deviations are denoted on the figure for each time point assessed.

The PK parameters for neratinib and everolimus were evaluated on cycle 1, day 15 using available samples obtained from eight patients who received study drugs at two different DLs, as summarized in Supplementary Table S5, available at https://doi.org/10.1016/j.esmoop.2025.104136. Additionally, the plasma concentration profiles are depicted in Figure 3. In the subgroup of patients (*n* = 5) administered neratinib 240 mg + everolimus 7.5 mg, the neratinib mean concentration exhibited a peak at 4.2 h, reaching a maximal concentration (C_max_) of 83.4 ng/ml, while the trough concentration (C_24_) was 44 ng/ml. The mean area under the concentration-time curve (AUC) was 1167 ng∗h/ml, mean half-life was 28.6 h, and the oral clearance was 289 l/h. The mean everolimus PK parameters were as follows: AUC, half-life, and oral clearance were 116.4 ng∗h/ml, 16.8 h, and 138 l/h, respectively. The mean time to maximum concentration was 4.8 h after dose administration with a mean C_max_ of 8.5 ng/ml and mean trough concentration of 2.8 ng/ml.

For the patients (*n* = 3) dosed with neratinib 200 mg + everolimus 5 mg, neratinib mean concentration peaked at 4.8 h with a C_max_ of 35.75 ng/ml and C_24_ of 13.6 ng/ml. The mean AUC was 399.7 ng∗h/ml, mean half-life was 13.1 h, and the oral clearance was 598 l/h. The mean everolimus PK parameters were as follows: AUC, half-life, and oral clearance were 24.3 ng∗h/ml, 8.2 h, and 202 l/h, respectively. The mean time to maximum concentration was 4.33 h after dose administration with a mean C_max_ of 3.58 ng/ml and a mean trough concentration of 0.42 ng/ml.

## Discussion

This study represents the first exploration of safety, toxicity, and preliminary antitumor efficacy in patients with advanced solid tumors treated with neratinib and everolimus. The toxicity profile of the combined therapy was found to be acceptable, which allowed dose escalation to the highest level (DL 5: neratinib 240 mg + everolimus 10 mg) until two DLTs (G3 mucositis and G3 diarrhea) were encountered. Following the standard 3 + 3 design, DL 4 (neratinib 240 mg + everolimus 7.5 mg) was expanded to include three additional patients for evaluation with one of six patients having DLT (G3 elevated creatinine). Consequently, DL 4 was identified as the MTD.

Previous studies have established the MTD of neratinib at 320 mg, while the MTD for everolimus remains undetermined, although PK analysis suggests 10 mg/day with acceptable tolerability.^35^, ^36^, ^37^ The predominant adverse events of neratinib monotherapy (240 mg) were diarrhea (100%), nausea (67%), fatigue (67%), and anorexia (67%), whereas rash (60.6%), nausea (42.4%), and mucositis (51.5%) were the common toxicities in single-agent everolimus (10 mg).^35^, ^36^, ^37^ Our study suggests that there may be additive toxicity of the combination therapy, with DLTs observed in two patients treated at DL 5 (neratinib 240 mg + everolimus 10 mg), which is below the MTD of single-agent neratinib. However, when reviewing DL 4 (neratinib 240 mg + everolimus 7.5 mg) which is our declared MTD, the toxicity profile mirrored previous studies, revealing that diarrhea (*n* = 8, 100%), aspartate aminotransferase elevation (*n* = 5, 62.5%), mucositis (*n* = 3, 37.5%), and nausea (*n* = 4, 50%) as the most prevalent TRAEs (Supplementary Tables S2 and S3, available at https://doi.org/10.1016/j.esmoop.2025.104136). Further analysis of the combination therapy toxicity profile, stratified by grades, however, reveals additional side-effects including an increased incidence of G3 diarrhea in both DL 4 and 5, and increased incidence of anemia, leukopenia, neutropenia, elevated liver enzymes, weight loss, creatinine elevation, hyponatremia, and lipid profile elevation (Supplementary Tables S6 and S7, available at https://doi.org/10.1016/j.esmoop.2025.104136), when compared with the comparable dose of single-agent neratinib or everolimus.

Based on the PK results from individual patients enrolled into DL 2 (neratinib 200 mg + everolimus 5 mg; *n* = 3) and DL 4 (neratinib 240 mg + everolimus 7.5 mg; *n* = 5), that we were able to fully assess the PK parameters, DL 2 exhibited neratinib PK parameters similar to those published for monotherapy.^35^^,^^38^ Interestingly, the neratinib PK parameters observed in the patients enrolled in DL 4 exhibited lowered neratinib clearance leading to a higher mean exposure of neratinib (C_max_, trough concentrations, and AUCs) and accumulation ratios. This is most likely due to saturation of the shared CYP3A4 enzymatic metabolic pathway with everolimus. Despite this, patients did not experience increased toxicity. For everolimus, based on the analyzed concentrations and PK in these two groups, the observed values and mean PK parameters were slightly lower than those reported for single-agent everolimus.^37^ This could be due, in part, to the high variability in absorption and the concomitant administration with neratinib, which led to the delayed time to maximal concentration. In both groups, the mean Tmax was over 4 h as compared with 1-2 h in prior reports.^36^^,^^37^ Despite these differences, the patients in DL 4 experienced higher exposure of everolimus in a non-linear fashion. However, this increased exposure did not contribute to increased toxicity.

In this study, the efficacy outcomes were improved compared with those observed in prior studies that focused solely on the use of neratinib as a monotherapy.^35^^,^^39^ Previous research reported overall response rates (ORR) ranging from 9.5% to 13.3% and clinical benefit rates (CBRs) between 23.8% and 25% across various tumor types.^35^^,^^39^ In contrast, our study demonstrated an ORR of 19% and a CBR of 28.6%, which are meaningful in a heavily pre-treated population with advanced cancers. Notably, two cases showed prolonged therapeutic responses. The first involved a 63-year-old female patient with cervical adenocarcinoma and ErbB2 overexpression identified by IHC, who achieved a best RECIST response of PR (−65%) by the end of cycle 6 and maintained this PR for >14 months. As of the last data analysis (31 July 2023), she remained on therapy and in PR. This extended response is particularly encouraging, suggesting that certain subgroups may derive benefit from this combination therapy. The second case involved a 41-year-old female with colorectal cancer and *ErbB*2 amplification (identified by FISH and NGS), who had previously received multiple lines of ErbB2-targeted therapies, including trastuzumab, pertuzumab, and a bispecific ErbB2 × 4-1BB agent. This patient achieved a PR (−30%) by cycle 6, day 26, and sustained the response for 66.3 weeks, with a deepening of the PR (−32%) at cycle 12, day 28.

However, the overall median duration of participation (3.2 months) reflects the challenging nature of this heavily pre-treated population. While some patients demonstrated significant and durable responses, the short median duration highlights that many may not experience long-term benefits. Future studies should aim to better identify predictive biomarkers and refine therapeutic strategies to enhance the durability of responses in this population.

The phase I neratinib monotherapy study by Wong et al. highlighted the effectiveness of targeted therapies for specific patient groups, notably breast and NSCLC patients previously treated with anti-ErbB2 and EGFR therapies.^35^ In their research, PRs were exclusively seen in breast cancer patients, with one instance of SD, while NSCLC patients exhibited six cases of SD.^35^ Our study, however, extends the efficacy of treatments to a wider array of cancers, including bladder, colorectal, and cervical, showcasing notable results. A critical distinction between our work and Wong et al.’s lies in the selection of participants. While they included patients with ErbB2 or EGFR-positive tumors identified via IHC only, our study broadened the criteria to include individuals with activating mutations, amplifications, and overexpression in *ErbB* family genes, using techniques such as FISH, IHC, and NGS. The treatment responses seen in our study, in cancer types beyond FDA-approved ErB2-positive breast cancer, suggests that ErbB2 is emerging as a potential target across tumor types.

Recent findings from the DESTINY-PanTumor01 study have highlighted the expanding potential of ErbB2-targeted therapies beyond breast cancer, demonstrating that trastuzumab deruxtecan shows significant efficacy across multiple solid tumors with activating *ErbB2* mutations. With an ORR of 29.4% and a disease control rate of 75.5% across various cancers, this study supports a broader application of ErbB2-directed treatments. Notably, responses were observed in patients with different mutation domains and even in those without ErbB2 overexpression, suggesting a pan-tumor applicability for ErbB2-targeted therapies.^40^

Similarly, our study illustrates the potential of combining neratinib with PI3K/mTOR pathway inhibition via everolimus, showing antitumor activity across a range of ErbB alterations. While our ORRs were modest in this heavily pre-treated cohort, the prolonged responses observed in certain subgroups of patients support the value of dual pathway inhibition. These findings suggest that ErbB and related pathways remain viable therapeutic targets across diverse tumor types and align with the broader shift toward ErbB2-targeted approaches seen in DESTINY-PanTumor01. Together, these results underscore the promise of the neratinib and everolimus combination, particularly for patients with treatment-resistant cancers.^40^

To better understand the impact of different types of ErbB alterations on treatment outcomes, we created an OncoPrint diagram from patients’ pre-existing molecular reports at study enrollment (Figure 2). The data revealed that among the patients, nine patients (41%) harbored *ErbB2* mutations, while eight (36.4%) had ErbB2 amplification or overexpression as determined by IHC. The mutation subgroup showed a modest improvement in CBR of 33%, with two PRs and one instance of SD, compared with a CBR of 25% in the ErbB2-amplified or -overexpressed group (two PRs). The small sample size and modest difference suggest that *ErbB2* mutations and amplification/overexpression by IHC are associated with comparable clinical benefits. Further investigation into co-occurring mutations is warranted to deepen our understanding of their role in treatment response. Additionally, the OncoPrint diagram demonstrated that 55% (*n* = 12) of patients exhibited *TP53* mutations, of which 75% were missense mutations. Our data are consistent with previous studies reporting *TP53* as the most frequently mutated tumor suppressor genes in cancers, with mutations present in 50% of human cancers and ∼80% being missense mutations.^41^

We observed antitumor effects across various dosages, paralleling the approach of Gandhi et al. with neratinib and temsirolimus.^42^ This suggests that lower doses of the drugs might be effective in achieving the desired therapeutic outcomes.^42^ Notably, Gandhi et al. reported a CBR of 71.4%, including 2 CRs, 6 PRs, and 27 cases of SD among 49 patients assessed for response. In our study, stratification by mutation type revealed that patients with concurrent *ErbB2/3* and *PI3K* pathway alterations (*n* = 9, 3 PRs, ORR 33.3%) exhibited a higher ORR compared with those with ErbB*2* alterations alone (*n* = 12, 1 PR, ORR 8.3%). This improved response was especially notable in rectal, bladder, and cervical tumors harboring PI3K/AKT/mTOR pathway alterations. The most significant tumor reduction of −65%, with the response still ongoing for more than a year after the data cut-off, was observed in a cervical cancer patient with ErbB2 overexpression and additional *PIK3CA, AKT*, and *mTOR* mutations (Figure 2). These findings align with the mechanistic actions of neratinib and everolimus.

In contrast, triple-negative breast cancer (TNBC) presents a distinct profile, characterized by the absence of estrogen receptor, progesterone receptor, and ErbB2 expression. TNBC frequently relies on alternative signaling pathways including Wnt/β-catenin, Notch, Hedgehog, transforming growth factor-β, and NF-kB, alongside the PI3K/AKT/mTOR pathway.^43^ This broader reliance on diverse pathways may reduce TNBC’s dependency on the ErbB2/PI3K/mTOR axis, potentially explaining the shorter, less-sustained response observed in the TNBC case with PR (−33%) and DOR of 12.7 weeks.

The difference in CBR between Gandhi et al. and our study could be partly attributed to the differences in patient demographics, particularly the proportion of patients with fewer prior systemic treatments (68.5% had three or more previous treatments, compared with 77% in our study) and a higher percentage of their patients were naïve to ErbB2-targeted therapy (76.7% with no prior ErbB2-targed therapies, compared with 41% in our study).^42^

We observed a CBR of 15.4% (one PR and one SD) in patients previously treated with anti-EGFR or anti-ErbB2 therapies in our study. In contrast, anti-EGFR and anti-ErbB2 treatment-naïve patients exhibited a higher CBR of 44.4% (three PRs and one SD). To evaluate the impact of prior treatment resistance on outcomes, we analyzed the number of previous therapy lines among patients who achieved clinical benefit versus those who did not. Our analysis indicates that non-responders (*n* = 16) had a higher median number of prior therapies (median = 5) compared with responders (*n* = 6, median = 3), with prior EGFR/ErbB2 therapy more prevalent in non-responders (68.8% versus 33.3%). These results suggest that fewer prior treatments and reduced EGFR/ErbB2 exposure may enhance responsiveness to the neratinib and everolimus combination, corroborating findings from a previous phase II study by Burstein et al., which demonstrated better efficacy of neratinib in patients without prior trastuzumab therapy.^44^ Interestingly, Sudhan et al. associated a lack of response to neratinib with gain-of-function alterations in the PIK3CA pathway and showed that combined therapy of neratinib with everolimus can overcome neratinib resistance.^25^ To determine whether our data align with their findings, we analyzed the subset of patients with co-occurring actionable alterations in ErbB and the PIK3CA pathway who had progressed on previous anti-ErbB2 therapy. Contrary to their findings, we did not see clinical benefit in these patients (*n* = 3), as all had discontinued study due to progressive disease, with a median treatment duration of 2 months. These findings, though from a small subset of patients, underscore the complexity of cancer signaling and the need for further exploration and understanding of the mechanism behind resistance to targeted agents. Insights gleaned from these studies would be vital to improve treatment outcomes.

In this article, we acknowledge limitations inherent in our study. Firstly, molecular testing was not carried out immediately before study entry, limiting our ability to utilize real-time biomarkers and molecular profiling to precisely target the core oncogenic signaling pathways active in the patients’ tumors. Additionally, ErBb2 overexpression was not centrally confirmed by IHC, which may have introduced variability in test results due to technical inconsistencies across different testing sites. As it stands, the molecular testing carried out historically on our patients revealed that most patients had co-occurring pathogenic mutations that are known to play key roles in oncogenesis. The genetic heterogeneity observed, with some patients harboring multiple mutations, adds complexity to the treatment landscape. This diversity may partly explain the variability in clinical outcomes, as certain alterations might confer greater sensitivity or resistance to the combination therapy.

Secondly, our small study population precludes drawing statically significant conclusions. Also, as a single-center study, potential biases related to patient selection and institutional practices may affect the broader applicability of our findings. Validation through larger, multicenter studies is needed to confirm and expand upon these results. Additionally, the heterogeneity of our patient population, with diverse tumor types, prior treatment histories, and various DL assignments could have significantly influenced the response to the intervention. Moreover, our patients were heavily pre-treated with a median of four prior lines of therapy (including prior EGFR and/or ErbB2-targeted therapies) which could have altered the genomic landscape of patients’ tumors. Finally, our OncoPrint diagram includes genetic alterations regardless of pathogenicity, offering a more comprehensive view of the spectrum of alterations among enrolled patients, with the aim of identifying potential biomarkers or the influence of variants of unknown significance (VUS) on therapeutic outcomes. However, VUS introduce uncertainty and complexity in interpreting genomic data for clinical application. Further research is needed to validate and elucidate the functional significance of VUS, which could ultimately expand therapeutic options and enhance precision in oncology.

The advanced stage of the enrolled patients suggests that the study was conducted in a difficult-to-treat population. The fact that 59.1% of patients had previously received EGFR/ErbB2-targeted therapies and had a median of four prior systemic therapies underscores the importance of identifying new effective treatment combinations for those who may have developed resistance to prior therapies. We believe that the CBR of 28.6% observed in this heavily pre-treated cohort is meaningful, highlighting the potential of the neratinib and everolimus combination to overcome resistance mechanisms and provide a therapeutic option for patients with limited alternatives.

In summary, the combination of neratinib and everolimus demonstrated a manageable safety profile and demonstrated signs of clinical efficacy, particularly in a heavily pre-treated population with ErbB2 alterations. While these findings are promising, further studies in larger cohorts are warranted to confirm efficacy and to identify specific biomarkers that may better predict patient response, thereby enhancing the precision of patient selection. Additionally, optimizing management strategies for common toxicities, such as diarrhea, could improve the regimen’s overall tolerability and support broader clinical application.

## Disclosure

**SAPP** reports clinical trial research funding/grant support through the institution from AbbVie, ABM Therapeutics, Acepodia, Alkermes, Aminex Therapeutics, Amphivena Therapeutics, BioMarin Pharmaceutical, Boehringer Ingelheim, Bristol Myers Squib, Cerulean Pharma, Chugai Pharmaceutical, Curis, Cyclacel Pharmaceuticals, Daiichi Sankyo, Eli Lilly, ENB Therapeutics, Epigenetix, Five Prime Therapeutics, F-Star Beta Limited, F-Star Therapeutics, Gene Quantum, Genmab A/S, Gilead Sciences, GlaxoSmithKline, Helix BioPharma, Hengrui Pharmaceuticals, HiberCell, Immorna Biotherapeutics, Immunomedics, Incyte, Jacobio Pharmaceuticals, Jiangsu Simcere Pharmaceutical, Lytix Biopharma AS; Medimmune, Medivation, Merck Sharp and Dohme Corp., Nectin Therapeutics, Novartis Pharmaceuticals, Pieris Pharmaceuticals, Pfizer, Phanes Therapeutics, Principia Biopharma, Puma Biotechnology, Purinomia Biotech, Rapt Therapeutics, Replimune, Seattle Genetics, Silverback Therapeutics, Synlogic Therapeutics, Taiho Oncology, Tesaro, Inc., TransThera Bio, ZielBio, NCI/NIH; P30CA016672—Core Grant (CCSG Shared Resources) and also worked as a consultant for CRC Oncology. **AN** reports research funding/grant support for clinical trials from NCI, EMD Serono, MedImmune, Healios Onc. Nutrition, Atterocor/Millendo, Amplimmune, ARMO BioSciences, Karyopharm Therapeutics, Incyte, Novartis, Regeneron, Merck, Bristol-Myers Squibb, Pfizer, CytomX Therapeutics, Neon Therapeutics, Calithera Biosciences, TopAlliance Biosciences, Eli Lilly, Kymab, PsiOxus, Arcus Biosciences, NeoImmuneTech, Immune-Onc Therapeutics, Surface Oncology, Monopteros Therapeutics, BioNTech SE, Seven & Eight Biopharma, and SOTIO Biotech AG; consulting fees from CTI, Deka Biosciences, Janssen Biotech, NGM Bio, PsiOxus Therapeutics, Immune-Onc Therapeutics, STCube Pharmaceuticals, OncoSec KEYNOTE-695, Genome & Company, CytomX Therapeutics, Nouscom, Merck Sharp & Dohme Corp, Servier, Lynx Health, AbbVie, and PsiOxus. AN received travel and accommodation expense from ARMO BioSciences, NeoImmuneTech, and NGM Biopharmaceuticals; and honoraria for speaking engagement from AKH Inc, The Lynx Group, Society for Immunotherapy of Cancer (SITC), Korean Society of Medical Oncology (KSMO), Scripps Cancer Care Symposium, ASCO Direct Oncology Highlights, European Society for Medical Oncology (ESMO), and CME Outfitters. **EED** reports research funding/grant support from Bayer HealthCare Pharmaceuticals Inc., Immunocore LTD, Amgen, Aileron Therapeutics, Compugen Ltd., TRACON Pharmaceuticals Inc., Unum Therapeutics, Gilead Immunomedics, BOLT Therapeutics, Aprea Therapeutics, Bellicum Pharmaceuticals, PMV Pharma, Triumvira Immunologics, Seagen Inc., Mereo BioPharma 5 Inc., Sanofi, Rain Oncology, Astex Therapeutics, Sotio, Poseida, Mersana Therapeutics, Genentech, Boehringer Ingelheim, Dragonfly Therapeutics, A2A Pharma, Volastra, and AstraZeneca; and serves in advisory board for BOLT Therapeutics, Mersana Therapeutics, Orum Therapeutics, Summit Therapeutics, PMV Pharma, and Fate Therapeutics. EED receives travel and accommodation expenses from BOLT Therapeutics, Mersana Therapeutics, Orum Therapeutics, Summit Therapeutics, PMV Pharma, and Fate Therapeutics; and serves as speaker for PMV Pharma. **KPR** reports research funding/grant support from AbbVie, AstraZeneca, Bayer, Daiichi Sankyo, Eisai, Genentech, Guardant Health, HiberCell, Innovent, Janssen, Merck, Seattle Genetics, UCB Biosciences, and Xencor; and serves as consultant for AstraZeneca, Bayer, Daiichi Sankyo, Eisai, Genentech, and Seattle Genetics. **FMB** reports research funding/grant support for clinical trials from Aileron Therapeutics, AstraZeneca, Bayer Healthcare Pharmaceutical, Calithera Biosciences, Curis, CytomX Therapeutics, Daiichi Sankyo, Debiopharm International, eFFECTOR Therapeutics, Genentech, Guardant Health, Klus Pharma, Takeda Pharmaceutical, Novartis, Puma Biotechnology, Taiho Pharmaceutical. FMB served in the advisory committee for Black Diamond, Biovica, Eisai, FogPharma, Immunomedics, Inflection Biosciences, Karyopharm Therapeutics, Loxo Oncology, Mersana Therapeutics, OnCusp Therapeutics, Puma Biotechnology Inc., Seattle Genetics, Sanofi, Silverback Therapeutics, Spectrum Pharmaceuticals, and Zentalis; received personal fees for consulting/travel related from AbbVie, Aduro BioTech, Alkermes, AstraZeneca, Daiichi Sankyo, DebioPharm, Ecor1 Capital, eFFECTOR Therapeutics, F. Hoffman-La Roche, GT Apeiron, Genentech, Harbinger Health, IBM Watson, Infinity Pharmaceuticals, Jackson Laboratory, Kolon Life Science, Lengo Therapeutics, Menarini Group, OrigiMed, PACT Pharma, Parexel International, Pfizer, Protai Bio, Samsung Bioepis, Seattle Genetics, Tallac Therapeutics, Tyra Biosciences, Xencor, Zymeworks, European Organization for Research and Treatment of Cancer (EORTC), and European Society for Medical Oncology (ESMO); and received personal fees for honoraria from Chugai Biopharmaceuticals**. HTT** serves as a consultant for ABION Bio**. PTS** reports research funding/grant support from GlaxoSmithKline, Novartis Oncology, Leap Therapeutics, and Merck. PTS served on an advisory board for GlaxoSmithKline and Aadi. All other authors have declared no conflicts of interest.
