# Supplementary material for: Phase I trial of the combination of the pan-ErbB inhibitor neratinib and mTOR inhibitor everolimus in advanced cancer patients with ErbB family gene alterations
Source: ESMO Open. 2025 Feb 4;10(2):104136. doi: 10.1016/j.esmoop.2025.104136 (PMC11847258; doi:10.1016/j.esmoop.2025.104136)
Supplement: Supplementary Figure 1 [file mmc1.docx]

**Supplementary Figure 1 Consort Flow Diagram**

Consented (n=39)

Excluded (n=17)

- Not meeting inclusion criteria (n=8)
- Declined to participate (n=3)
- Other reasons (n=6)

Enrollment

Assessed for eligibility (n=39)

Patient included in safety response analysis (n=22)

Efficacy (n=21)

Excluded for new brain metastasis (n=1)
