# Supplementary material for: Phase I trial of the combination of the pan-ErbB inhibitor neratinib and mTOR inhibitor everolimus in advanced cancer patients with ErbB family gene alterations
Source: ESMO Open. 2025 Feb 4;10(2):104136. doi: 10.1016/j.esmoop.2025.104136 (PMC11847258; doi:10.1016/j.esmoop.2025.104136)
Supplement: Supplementary Table 1 [file mmc2.docx]

**Supplementary Table** **S1**: Clinical Overview of ErbB alteration Identification and Prior Therapy

| **Disease type** | **ErbB alteration identification/ Date** | **First ErbB2 therapy** | **Lines before enrolling** | **No. of systemic therapy before ErbB2/EGFR therapies** | **Prior PI3K/AKT/mTOR** | **Clinical Benefit** |
| --- | --- | --- | --- | --- | --- | --- |
| Breast | FISH / (4/23/2015) | 5/unk/2015 | 7 | 2 | No | No |
| Esophageal | IHC 3+/ (8/1/2016) | 9/13/2016 | 1 | 0 | No | Yes |
| Hemangiopericytoma | NGS (2/23/2016) | 4/18/2017 | 3 | 2 | No | No |
| Breast | NGS (6/20/2018) | N/A | 2 | N/A | No | Yes |
| Colorectal | NGS (6/6/2016) | N/A | 4 | N/A | No | No |
| Bladder | NGS (5/18/2017) | N/A | 4 | N/A | No | Yes |
| Breast | IHC 3+ (6/16/2008)  (6/10/2011) | 8/25/2008 | 11 | 1 | No | No |
| Breast | NGS-EGFR (10/12/2017) | N/A | 9 | N/A | Yes | No |
| Colorectal | IHC 3+ (4/21/2014)  NGS (4/14/2016)  FISH (1/26/2017) | 3/8/2018 | 5 | 3 | No | Yes |
| Ovarian | NGS 1/12/2018 | 1/29/2019 | 3 | 2 | No | No |
| Colorectal | NGS (6/1/2017)  FISH (7/6/2017) | 1/24/2019 | 4 | 2 | No | No |
| Cervical | NGS 2/1/2017 | N/A | 6 | N/A | No | No |
| Endometrial | NGS 2/12/2019 | N/A | 3 | N/A | No | No |
| Breast | NGS 10/10/2019 | N/A | 10 | N/A | Yes | No |
| Lung | NGS 1/19/2016 | 6/8/2018 | 6 | 2 | No | No |
| Endometrial | IHC3+ (6/8/2020)  NGS (1/27/2021) | 6/25/2020 | 2 | 0 | No | No |
| Cervical | IHC 3+ (7/3/2019) | N/A | 2 | N/A | No | Yes |
| Colorectal | FISH (9/13/2019)  IHC (9/13/2019)  NGS (1/5/2021) | 3/2/2021 | 11 | 1 | No | No |
| Parotid gland | NGS 1/27/2021 | 10/14/2021 | 4 | 2 | No | No |
| Breast | NGS 10/22/2021 | 8/26/2022 | 7 | 6 | No | No |
| Breast | NGS 9/17/2020 | N/A | 8 | N/A | No | Yes |
| Unknown primary | NGS 10/21/2019 | 7/31/2021 | 2 | 1 | No | No |

Abbreviations: FISH, fluorescence in situ hybridization; IHC, immunohistochemistry; NGS, next generation sequencing; N/A, not applicable; unk, unknown.
