# Supplementary material for: Phase I trial of the combination of the pan-ErbB inhibitor neratinib and mTOR inhibitor everolimus in advanced cancer patients with ErbB family gene alterations
Source: ESMO Open. 2025 Feb 4;10(2):104136. doi: 10.1016/j.esmoop.2025.104136 (PMC11847258; doi:10.1016/j.esmoop.2025.104136)
Supplement: Supplementary Table 2 [file mmc3.docx]

**Supplementary Table S2:** **Neratinib and everolimus safety assessment**

| Dose Level  Neratinib  Everolimus | 1  160mg  5mg | 2  200mg  5mg | 3  200mg  7.5mg | 4  240mg  7.5mg | 5  240mg  10mg | All |
| --- | --- | --- | --- | --- | --- | --- |
| **Safety assessment** | N=5 | N=4 | N=3 | N=8 | N=2 | N=22 |
| DLT, n (%) |  |  |  |  |  |  |
|  | 0 | 0 | 0 | 1 (12.5) | 2 (100) | 3 (13.6) |
| TRAE, n (%) |  |  |  |  |  |  |
| ≥ 1 TRAE | 5 (100.0) | 3 (75.0) | 3 (100) | 8 (100) | 2 (100) | 21 (95.4) |
| ≥ 1 TRAE grade ≥ 3 | 0 | 1 (25.0) | 0 | 3 (37.5) | 2 (100) | 6 (27.2) |
| ≥ 1 TRAE grade ≥ 3 |  |  |  |  |  |  |
|  |  |  |  |  |  |  |
| ≥ 1 SAE related to neratinib | 0 | 1 (25.0) | 0 | 0 | 1 (50.0) | 2 (9.1) |
| ≥ 1 SAE related to everolimus | 0 | 0 | 0 | 0 | 0 | 0 |
| Treatment discontinuation, n (%) |  |  |  |  |  |  |
| ≥ 1 TRAE resulting in discontinuation | 0 | 1 (25.0) | 0 | 0 | 0 | 1 (4.5) |
| PD  Clinical PD  Other | 4 (80.0)  0  1 (20.0)* | 2 (50.0)  1 (25.0)  0 | 3 (100)  0  0 | 5 (62.5)  2 (25)  1 (12.5) ^ | 2 (100)  0  0 | 16 (72.7)  3 (13.6)  2 (9.1) |
|  |  |  |  |  |  |  |

Abbreviations: N, number; D, day; Ner, neratinib; Eve, everolimus; DLT, dose-limiting toxicity, TRAE treatment-related adverse event, SAE serious adverse event; PD, progressive disease; mg, milligram. Symbols: * not meeting protocol criteria; ^ remains active in study.
