# Supplementary material for: Phase I trial of the combination of the pan-ErbB inhibitor neratinib and mTOR inhibitor everolimus in advanced cancer patients with ErbB family gene alterations
Source: ESMO Open. 2025 Feb 4;10(2):104136. doi: 10.1016/j.esmoop.2025.104136 (PMC11847258; doi:10.1016/j.esmoop.2025.104136)
Supplement: Supplementary Table 3 [file mmc4.docx]

**Supplementary Table S3: Treatment-related adverse events (TRAEs) for neratinib and everolimus**

| Adverse event  Number (%)* | **NER 160mg**  **Eve 5mg** | | **NER 200mg**  **Eve 5mg** | | **NER 200mg**  **Eve 7.5mg** | | **NER 240mg**  **Eve 7.5mg** | | **NER 240mg**  **Eve 10mg** | | **All** | |
| --- | --- | --- | --- | --- | --- | --- | --- | --- | --- | --- | --- | --- |
|  | **N=5** | | **N=4** | | **N=3** | | **N=8** | | **N=2** | | **N=22** | |
|  | **G<3** | **G3** | **G<3** | **G3** | **G<3** | **G3** | **G<3** | **G3** | **G<3** | **G3** | **G<3** | **G3** |
| Diarrhea | 0 | 0 | 2  (50) | 1  (25) | 2  (66.6) | 0 | 8  (100) | 1  (12.5) | 2  (100) | 2  (100) | 14  (63.6) | 4  (18.2) |
| Cough | 0 | 0 | 1  (25) | 0 | 0 | 0 | 0 | 0 | 0 | 0 | 1  (4.5) | 0 |
| Neutropenia | 1  (20) | 0 | 0 | 0 | 0 | 0 | 2  (25) | 0 | 0 | 0 | 3  (13.6) | 0 |
| Thrombocytopenia | 2  (40) | 0 | 0 | 0 | 1  (33.3) | 0 | 1  (12.5) | 0 | 1  (50) | 0 | 5  (22.7) | 0 |
| Confusion | 0 | 0 | 0 | 0 | 0 | 0 | 0 | 0 | 1  (50) | 0 | 1  (4.5) | 0 |
| Nausea | 1  (20) | 0 | 1  (25) | 0 | 1  (33.3) | 0 | 4  (50) | 0 | 0 | 0 | 7  (31.8) | 0 |
| Anemia | 0 | 0 | 1  (25) | 0 | 0 | 0 | 3  (37.5) | 2  (25) | 0 | 0 | 4  (18.2) | 2  (9.1) |
| Leukopenia | 1  (20) | 0 | 0 | 0 | 0 | 0 | 2  (25) | 0 | 0 | 0 | 3  (13.6) | 0 |
| Mucositis | 0 | 0 | 2  (50) | 1  (25) | 2  (66.6) | 0 | 3  (37.5) | 0 | 2  (100) | 1  (50) | 9  (41) | 2  (9.1) |
| Stomatitis | 1  (20) | 0 | 0 | 0 | 0 | 0 | 0 | 0 | 0 | 0 | 1  (4.5) | 0 |
| Anorexia | 1  (20) | 0 | 1  (25) | 0 | 0 | 0 | 2  (25) | 0 | 0 | 0 | 4  (18.2) | 0 |
| Rash | 0 | 0 | 1  (25) | 0 | 1  (33.3) | 0 | 1  (12.5) | 0 | 1  (50) |  | 4  (18.2) | 0 |
| Vomiting | 0 | 0 | 0 | 0 | 1  (33.3) | 0 | 2  (25) | 0 | 0 | 0 | 3  (13.6) | 0 |
| Albumin decreased | 0 | 0 | 0 | 0 | 0 | 0 | 1  (12.5) | 0 | 0 | 0 | 1  (4.5) | 0 |
| Hyperglycemia | 1  (20) | 0 | 1  (25) | 0 | 1  (33.3) | 0 | 1  (12.5) | 0 | 0 | 0 | 4  (18.2) | 0 |
| Hypophosphatemia | 0 | 0 | 0 | 0 | 0 | 0 | 1  (12.5) | 0 | 0 | 0 | 1  (4.5) | 0 |
| Hypercholesterolemia | 0 | 0 | 1  (25) | 0 | 1  (33.3) | 0 | 2  (25) | 0 | 0 | 0 | 4  (18.2) | 0 |
| Hypokalemia | 0 | 0 | 0 | 0 | 0 | 0 | 1  (12.5) | 0 | 0 | 0 | 1  (4.5) | 0 |
| Weight loss | 0 | 0 | 0 | 0 | 1  (33.3) | 0 | 2  (25) | 0 | 0 | 0 | 3  (13.6) | 0 |
| AST elevation | 0 | 0 | 0 | 0 | 1  (33.3) | 0 | 5  (62.5) | 1  (12.5) | 0 | 0 | 6  (27.3) | 1  (4.5) |
| ALT elevation | 0 | 0 | 0 | 0 | 1  (33.3) | 0 | 3  (37.5) | 0 | 0 | 1  (50) | 4  (18.2) | 1  (4.5) |
| Acute Kidney Injury | 0 | 0 | 0 | 0 | 0 | 0 | 0 | 1  (12.5) | 0 | 1  (50) | 0 | 2  (9.1) |
| Creatinine increased | 1  (20) | 0 | 0 | 0 | 0 | 0 | 2  (25) | 1  (12.5) | 0 | 0 | 3  (13.6) | 1  (4.5) |
| Pneumonitis | 0 | 0 | 1  (25) | 0 | 0 | 0 | 0 | 0 | 0 | 0 | 1  (4.5) | 0 |
| Fatigue | 2  (40) | 0 | 0 | 0 | 0 | 0 | 1  (12.5) | 0 | 0 | 0 | 3  (13.6) | 0 |
| Hypertriglyceridemia | 5  (100) | 0 | 1  (25) | 0 | 2  (66.6) | 0 | 3  (37.5) | 0 | 0 | 0 | 11  (50.0) | 0 |
| Alkaline phosphatase increased | 0 | 0 | 0 | 0 | 0 | 0 | 1  (12.5) | 1  (12.5) | 0 | 0 | 1  (4.5) | 1  (4.5) |
| Dyspnea | 0 | 0 | 0 | 0 | 0 | 0 | 1  (12.5) | 0 | 0 | 0 | 1  (4.5) | 0 |
| Hypomagnesemia | 0 | 0 | 0 | 0 | 0 | 0 | 2  (25) | 0 | 0 | 0 | 2  (9.1) | 0 |
| Hypercalcemia | 0 | 0 | 0 | 0 | 0 | 0 | 1  (12.5) | 0 | 0 | 0 | 1  (4.5) | 0 |
| Hyponatremia | 0 | 0 | 0 | 0 | 0 | 0 | 2  (25) | 0 | 0 | 0 | 2  (9.1) | 0 |
| Neuropathy | 0 | 0 | 0 | 0 | 0 | 0 | 1  (12.5) | 0 | 0 | 0 | 1  (4.5) | 0 |
| Tachycardia | 0 | 0 | 0 | 0 | 0 | 0 | 1  (12.5) | 0 | 0 | 0 | 1  (4.5) | 0 |
| Investigation, other | 0 | 0 | 0 | 0 | 0 | 0 | 0 | 1 (12.5) | 0 | 0 | 0 | 1  (4.5) |

*The numbers represent the highest grades assigned. No patients experienced grade 4 or higher TRAE on study. Adverse events deemed at least possibly, probably, or definitely related to treatment were graded based on Common Terminology Criteria for Adverse Events, Version 4 (CTCAE 4.0)

Abbreviations: G, grade; N, number; Ner, neratinib; Eve, everolimus; AST, aspartate aminotransferase; ALT, alanine transaminase; mg, milligram.
