# Supplementary material for: Phase I trial of the combination of the pan-ErbB inhibitor neratinib and mTOR inhibitor everolimus in advanced cancer patients with ErbB family gene alterations
Source: ESMO Open. 2025 Feb 4;10(2):104136. doi: 10.1016/j.esmoop.2025.104136 (PMC11847258; doi:10.1016/j.esmoop.2025.104136)
Supplement: Supplementary Table 4 [file mmc5.docx]

| **Supplementary Table** **S4**: Severe Adverse Events (SAEs) | | | | | | | | | | |  |  |  |  |  |
| --- | --- | --- | --- | --- | --- | --- | --- | --- | --- | --- | --- | --- | --- | --- | --- |
| Dose Level | 1 | | | 2 | | 3 | | 4 | | 5 | | | |  | |
| Severe Adverse Events (SAEs) | NER 160 mg + Eve 5mg (N=5) | | | NER 200 mg + Eve 5mg (N=4) | | NER 200 mg + Eve 7.5 mg (N=3) | | NER 240 mg + Eve 7.5 mg (N=8)^¶^ | | NER 240 mg + Eve 10mg (N=2) | | | | All (N=22) | |
|  | G < 3 | ≥G3 | | G < 3 | ≥G3 | G < 3 | ≥G3 | G <3 | ≥G3 | G < 3 | | | ≥G3 | G < 3 | ≥G3 |
| Abdominal pain | 0 | | 0 | 0 | 0 | 0 | 0 | 0 | 1 (12.5) | 0 | | 0 | | 0 | 1 (4.5) |
| Creatinine increased | 0 | | 0 | 0 | 0 | 0 | 0 | 1 (12.5)*^ | 1 (12.5)*^ | 0 | | 0 | | 1 (4.5)*^ | 1 (4.5)*^ |
| Colonic stenosis | 0 | | 0 | 0 | 0 | 0 | 0 | 0 | 1(12.5) | 0 | | 0 | | 0 | 1 (4.5) |
| Diarrhea | 0 | | 0 | 0 | 1 (25)^*^ | 0 | 0 | 0 | 0 | 0 | | 1 (50)^*^ ^∆^ | | 0 | 2 (9.1)^*^ |
| Dyspnea | 0 | | 0 | 0 | 0 | 0 | 0 | 0 | 0 | 0 | | 1 (50) | | 0 | 1 (4.5) |
| Death | 0 | | 0 | 0 | 0 | 0 | 0 | 0 | 1 (12.5) | 0 | | 0 | | 0 | 1 (4.5) |
| Pericardial effusion | 0 | | 0 | 0 | 1 (25) | 0 | 0 | 0 | 0 | 0 | | 0 | | 0 | 1 (4.5) |
| Investigation, other | 0 | | 0 | 0 | 0 | 0 | 0 | 0 | 1 (12.5)*^ | 0 | | 0 | | 0 | 1 (4.5)*^ |
| UTI / Delirium | 0 | | 0 | 0 | 0 | 0 | 0 | 0 | 1 (12.5) | 0 | | 0 | | 0 | 1 (4.5) |
| Anemia/ Confusion | 0 | | 0 | 0 | 0 | 0 | 0 | 0 | 1 (12.5) | 0 | | 0 | | 0 | 1 (4.5) |
| UTI / AKI/ elevated liver enzymes/ Anemia | 0 | | 0 | 0 | 0 | 0 | 0 | 0 | 1 (12.5) | 0 | | 0 | | 0 | 1 (4.5) |
| Cholecystitis | 0 | | 0 | 0 | 0 | 0 | 0 | 0 | 1 (12.5) | 0 | | 0 | | 0 | 1 (4.5) |
| Rectal hemorrhage | 0 | | 0 | 0 | 0 | 0 | 0 | 0 | 1 (12.5) | 0 | | 0 | | 0 | 1 (4.5) |
| Pain | 0 | | 0 | 0 | 0 | 0 | 0 | 0 | 1 (12.5) | 0 | | 0 | | 0 | 1 (4.5) |

Abbreviations - N: number of patients, NER: neratinib, Eve: everolimus, G: grade, UTI: urinary tract infection, AKI: acute kidney injury

* Possibly related to neratinib

^ Possibly related to everolimus

∆ was defined as a dose-limiting toxicity.

¶ Dose level 4: (1 patient remains active on treatment as of data cut off on July 31^st^, 2023)

Adverse events were graded based on the Common terminology Criteria for Adverse Event, version 4.0 (CTCAE 4.0).
