# Supplementary material for: Phase I trial of the combination of the pan-ErbB inhibitor neratinib and mTOR inhibitor everolimus in advanced cancer patients with ErbB family gene alterations
Source: ESMO Open. 2025 Feb 4;10(2):104136. doi: 10.1016/j.esmoop.2025.104136 (PMC11847258; doi:10.1016/j.esmoop.2025.104136)
Supplement: Supplementary Table 5 [file mmc6.docx]

**Supplementary Table S5:** Neratinib and everolimus pharmacokinetic (PK) parameters on Cycle 1, Day 15

| **PK parameters** | **Dose Level 2** | |  | **Dose Level 4** | |
| --- | --- | --- | --- | --- | --- |
|  | **Neratinib** | **Everolimus** |  | **Neratinib** | **Everolimus** |
|  | **200mg/day, n=3** | **5mg/day n=3** |  | **240mg/day, n=5** | **7.5mg/day, n=5** |
| **Half-life (h)** | 13.1 (9.5 - 23.6) | 8.2 (5.5-10.9) |  | 28.6 (13.5-55.3) | 16.8 (3.3-33.7) |
| **Tmax (h)** | 4.8 (1 - 6) | 4.33 (1-8) |  | 4.2 (2-6) | 4.8 (4-6) |
| **Cmax (ng/mL)** | 35.75 (11.7 – 53.7) | 3.58 (1.3-5.1) |  | 83.4 (22.3-154) | 8.5 (2.5-14.6) |
| **AUC_0-24_ (ng*h/mL)** | 399.7 (81.9-1046) | 24.3 (14.2-29.5) |  | 1167 (347-3010) | 116.4 (12-281) |
| **AUC_0-∞_ (ng*h/mL)** | 910 (195-2125) | 53.8 (31-76.3) |  | 3932 (3031-1222) | 253 (16.9-762) |
| **Ctau or C_24_ ng/mL** | 13.6 (2.6-31.8) | 0.42 (0-1.1) |  | 44 (14.5-125) | 2.8 (0-9.9) |
| **CLss (L/h)** | 598 (191-1261) | 202 (985-352) |  | 289 (135-797) | 138 (37-368) |
| **AR** | 1.41(1.2-1.9) | 1.2 (1.1-1.3) |  | 2.29 (1.4-3.8) | 1.7 (1-2.6) |

All values are calculated as mean (range)

Abbreviations: Cmax, maximum plasma concentration; Tmax, time to achieve Cmax; AUC_0-24_, area under the curve time 0 to 24 h; AUC_0-∞_, area under the curve time 0 to infinity; Ctau, concentration at the end dosing interval; CLss, clearance at steady state; AR, accumulation ratio.
