# Supplementary material for: Phase I trial of the combination of the pan-ErbB inhibitor neratinib and mTOR inhibitor everolimus in advanced cancer patients with ErbB family gene alterations
Source: ESMO Open. 2025 Feb 4;10(2):104136. doi: 10.1016/j.esmoop.2025.104136 (PMC11847258; doi:10.1016/j.esmoop.2025.104136)
Supplement: Supplementary Table 6 [file mmc7.docx]

**Supplementary Table S6**: Common treatment-related adverse events of all grades (frequency> 20%) between combination and monotherapies of neratinib and everolimus

|  | Neratinib (240mg) + Everolimus (7.5mg)  (N=8 | Neratinib (240mg) + Everolimus (10mg)  (N=2) | Neratinib (240mg)  (N=3)  (Wong et.al.) | Everolimus  O’Donnell et.al. | |
| --- | --- | --- | --- | --- | --- |
|  |  |  |  | 5mg  (N=4) | 10mg  (N=33) |
| Diarrhea | 8 (100) | 2 (100) | 3 (100) | - | 7 (21.2) |
| Nausea | 4 (50) | - | 2 (67) | 1 (25) | 14 (42.4) |
| Vomiting | 2 (25) | - | 1 (33) | - | 5 (15.2) |
| Rash | - | 1 (50) | 1 (33) | 2 (50) | 20 (60.6) |
| Fatigue | - | - | 2 (67) | - | 12 (36.4) |
| Mucositis | 3 (37.5) | 2 (100) | - | 2 (50) | 17 (51.5) |
| Anorexia | 2 (25) | - | 2 (67) | 1 (25) | 11 (33.3) |
| Headache | - | - | - | - | 7 (21.2) |
| Constipation | - | - | - | 1 (25) | 7 (21.2) |
| Anemia | 3 (37.5) | - | - | 1 (25) | - |
| Leukopenia | 2 (25) | - | - | - | - |
| AST elevation | 5 (62.5) | - | - | - | - |
| ALT elevation | 3 (37.5) | 1 (50) | - | - | - |
| Pruritus | - | - | - | 1 (25) | - |
| Herpes simplex infection | - | - | - | 1 (25) | - |
| Confusion | - | 1 (50) | - | - | - |
| Thrombocytopenia | - | 1 (50) | - | - | - |
| Acute Kidney Injury | - | 1 (50) | - | - | - |
| Weight loss | 2 (25) | - | - | - | - |
| Creatinine Increased | 2 (25) | - | - | - | - |
| Hyponatremia | 2 (25) | - | - | - | - |
| Hypomagnesemia | 2 (25) | - | - | - | - |
| Neutropenia | 2 (25) | - | - | - | - |
| Hypercholesterolemia | 2 (25) | - | - | - | - |
| Hypertriglyceridemia | 3 (37.5) |  |  |  |  |
|  |  |  |  |  |  |

Abbreviations: N, number; AST, aspartate aminotransferase; ALT, alanine transaminase; mg, milligram.
