# Supplementary material for: Phase I trial of the combination of the pan-ErbB inhibitor neratinib and mTOR inhibitor everolimus in advanced cancer patients with ErbB family gene alterations
Source: ESMO Open. 2025 Feb 4;10(2):104136. doi: 10.1016/j.esmoop.2025.104136 (PMC11847258; doi:10.1016/j.esmoop.2025.104136)
Supplement: Supplementary Table 7 [file mmc8.docx]

**Supplementary Table** **S7**: Grade ≥ 3 treatment-related between combination and monotherapies of neratinib and everolimus

|  | Neratinib (240mg) + Everolimus (7.5mg)  (N=8 | Neratinib (240mg) + Everolimus (10mg)  (N=2) | Neratinib (240mg)  (N=3)  (Wong et.al.) | Everolimus  O’Donnell et.al. | |
| --- | --- | --- | --- | --- | --- |
|  |  |  |  | 5mg  (N=4) | 10mg  (N=33) |
| Diarrhea | 1 (12.5) | 2 (100) | - | - | - |
| Nausea |  | - | - | - | 1 (3) |
| Epistaxis | - | - | - | - | 1 (3) |
| Rash | - | - | - | - | 1 (3) |
| Anemia | 2 (25) | - | - | - | - |
| Mucositis | - | 1 (50) | - | - | 2 (6.1) |
| Anorexia | - | - | - | - | 2 (6.1) |
| Dyspnea | - | - | - | - | 1 (3) |
| GI hemorrhage | - | - | - | - | 1 (3) |
| Pneumonia | - | - | - | - | 1 (3) |
| Thrombocytopenia | - | - | - | - | 2 (6.1) |
| Hyperglycemia | - | - | - | - | 2 (6.1) |
| Hypertriglyceridemia | - | - | - | - | 1 (3) |
| Creatinine increased | 1 (12.5) | - | - | - | - |
| ALT elevation | - | 1 (50) | - | - |  |
| AST elevation | 1 (12.5) | - | - | - | - |
| Acute Kidney injury | 1 (12.5) | 1 (50) | - | - | - |
| Alkaline phosphatase increased | 1 (12.5) | - | - | - | - |
|  |  |  |  |  |  |
|  |  |  |  |  |  |
